# Cyclooxygenase-2 overexpression correlates with tumour recurrence, especially haematogenous metastasis, of colorectal cancer

**DOI:** 10.1054/bjoc.2000.1270

**Published:** 2000-07-03

**Authors:** S Tomozawa, N H Tsuno, E Sunami, K Hatano, J Kitayama, T Osada, S Saito, T Tsuruo, Y Shibata, H Nagawa

**Affiliations:** Department of Surgical Oncology, Graduate School of Medical Sciences; Department of Transfusion Medicine, University of Tokyo, 7-3-1 Hongo, Tokyo, Bunkyo-ku, 113-8655, Japan; Institute of Molecular and Cellular Biosciences, University of Tokyo, 1-1-1 Yayoi, Tokyo, Bunkyo-ku, 113-0032, Japan

**Keywords:** colorectal cancer, COX-2, immunohistochemistry, haematogenous metastasis, recurrence

## Abstract

Epidemiological studies have demonstrated that nonsteroidal anti-inflammatory drugs (NSAIDs), known to inhibit cyclooxygenase (COX), reduce the risk of colorectal cancer. COX is a key enzyme in prostaglandin biosynthesis, and two isoforms of COX, COX-1 and COX-2, have been identified. Recently COX-2 has been reported to frequently overexpress in colorectal neoplasms and to play a role in colorectal tumorigenesis and tumour progression. In this study, using immunohistochemistry, we examined COX-2 expression in advanced human colorectal cancer and its correlation with clinicopathological features. COX-2 expression was observed mainly in the cytoplasm of cancer cells in all the specimens examined, but some stromal cells and endothelial cells were also stained. According to the grade of COX-2 expression of the cancer cells, patients were divided into high- and low-COX-2 expression groups. High-COX-2 expression significantly correlated with tumour recurrence, especially haematogenous metastasis. These results suggest that a selective COX-2 inhibitor can be a novel class of therapeutic agents not only for tumorigenesis but also for haematogenous metastasis of cololectal cancer. To our knowledge, this is the first report on the correlation between COX-2 overexpression and recurrence of colorectal cancer. © 2000 Cancer Research Campaign


*British Journal of Cancer* (2000) **83**(3), 324–328
© 2000 Cancer Research Campaign
doi: 10.1054/ bjoc.2000.1270, available online at http://www.idealibrary.com on IDEAL®

### Cyclooxygenase-2 overexpression correlates with tumour recurrence, especially haematogenous metastasis, of colorectal cancer

**S Tomozawa[1], NH Tsuno[2], E Sunami[1], K Hatano[1], J Kitayama[1], T Osada[2], S Saito[1], T Tsuruo[3], Y Shibata[2] and H Nagawa[1]**

[1]Department of Surgical Oncology, Graduate School of Medical Sciences and [2]Department of Transfusion Medicine, University of Tokyo, 7-3-1 Hongo, Bunkyo-ku, Tokyo 113-8655, Japan; [3]Institute of Molecular and Cellular Biosciences, University of Tokyo, 1-1-1 Yayoi, Bunkyo-ku, Tokyo 113-0032, Japan

**Summary** Epidemiological studies have demonstrated that nonsteroidal anti-inflammatory drugs (NSAIDs), known to inhibit cyclooxygenase (COX), reduce the risk of colorectal cancer. COX is a key enzyme in prostaglandin biosynthesis, and two isoforms of COX, COX-1 and COX-2, have been identified. Recently COX-2 has been reported to frequently overexpress in colorectal neoplasms and to play a role in colorectal tumorigenesis and tumour progression. In this study, using immunohistochemistry, we examined COX-2 expression in advanced human colorectal cancer and its correlation with clinicopathological features. COX-2 expression was observed mainly in the cytoplasm of cancer cells in all the specimens examined, but some stromal cells and endothelial cells were also stained. According to the grade of COX-2 expression of the cancer cells, patients were divided into high- and low-COX-2 expression groups. High-COX-2 expression significantly correlated with tumour recurrence, especially haematogenous metastasis. These results suggest that a selective COX-2 inhibitor can be a novel class of therapeutic agents not only for tumorigenesis but also for haematogenous metastasis of cololectal cancer. To our knowledge, this is the first report on the correlation between COX-2 overexpression and recurrence of colorectal cancer. © 2000 Cancer Research Campaign

**Keywords**: colorectal cancer; COX-2; immunohistochemistry; haematogenous metastasis; recurrence

### INTRODUCTION

Several recent studies have reported a 40–50% lower risk of colorectal cancer in people who are continuously taking aspirin or other nonsteroidal anti-inflammatory drugs (NSAIDs) (Thun et al, 1991; 1993; Marnett, 1992; 1995; Giovannucci et al, 1994; 1995). The mechanism by which NSAIDs prevent colorectal carcinogenesis is not clear, but one possibility involves inhibition of cyclooxygenase (COX).

COX is a key enzyme in the conversion of arachidonic acid to prostaglandins, and two isoforms of COX, namely COX-1 and COX-2, have been identified. COX-1 is constitutively expressed in most cells and tissues, including normal intestinal and gastric mucosa (Williams and DuBois, 1996), and is considered to be involved in various physiological functions such as cytoprotection of the gastric mucosa. On the other hand, COX-2 is induced by a variety of agents including cytokines, hormones, growth factors, and tumour promoters, and its expression is elevated in inflammatory cells and sites of inflammation (Kujubu et al, 1991; O'Banion et al, 1991; Jones et al, 1993; DuBois et al, 1994; Xie and Hershman, 1995). Recently multiple studies have shown that COX-2 is expressed at high levels in 80–90% of colorectal adenocarcinomas (Eberhart et al, 1994; Kargman et al, 1995; Sano et al, 1995), and selective inhibition of COX-2 reduces colorectal tumourigenesis in different models of carcinogenesis (Oshima et al, 1996; Reddy et al, 1996; Kawamori et al, 1998). Thus, COX-2 has been thought to play a causal role in colorectal tumorigenesis.

Furthermore, it has recently been reported that a human colon cancer cell line transfected with a COX-2 expression vector acquired increased invasiveness with activation of matrix metalloproteinase (Tsuji et al, 1997). We also have shown that a selective COX-2 inhibitor, JTE-522, suppressed haematogenous metastasis of colon cancer in mice (Tomozawa et al, 1999). These findings suggest that COX-2 might be involved in not only tumorigenesis but also cancer metastasis.

Here, we examined COX-2 expression in primary advanced colorectal carcinoma tissue using immunohistochemistry and its correlation with clinicopathological features.

### MATERIALS AND METHODS

### Patient samples

Specimens from 63 patients with advanced colorectal cancer without haematogenous metastasis receiving radical surgical treatment at the Department of Surgical Oncology, the University of Tokyo, in the period 1990–1994, were evaluated. In all these patients, the depth of tumour invasion was proper muscle (mp) or subserosa (ss). The median follow-up period was 60 months (range 6–98).

### Immunohistochemistry

Tissue samples were fixed with 10% formaldehyde in phosphate-buffered saline (PBS), embedded in paraffin, and cut into

*Received 23 August 1999*
*Revised 14 February 2000*
*Accepted 16 March 2000*

*Correspondence to:* S Tomozawa

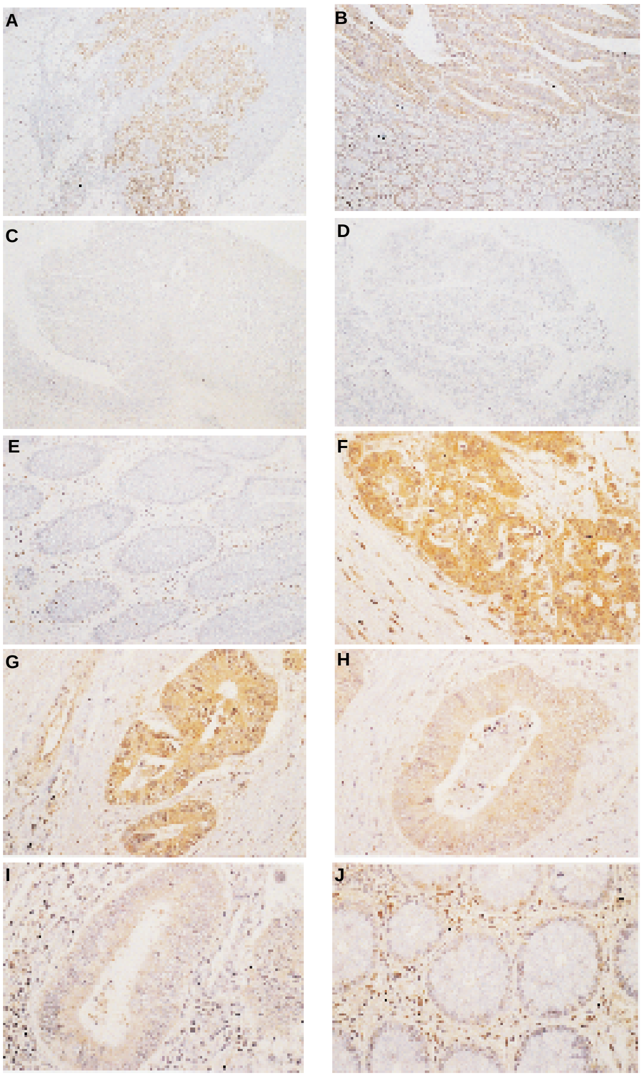

**Figure 1** COX-2 immunostaining (**A**, **B**, **F**, **G**, **H**, **I**, **J**). Negative control (**C**). COX-1 immunostaining (**D**, **E**). (**A**) (× 25) A representative case of colon cancer tissue (grade 3). COX-2 expression is observed in the cytoplasm and in the nuclear envelope of the tumour cells, and some stromal cells are stained. The extent of immunostaining of the tumour is diffuse. (**B**) (× 25) A large field of view encompassing tumour and adjacent tissue. Immunostaining of the cancer tissue is stronger than the normal epithelium. (**C**) (× 10) A negative control by non-immunized rabbit serum. (**D** and **E**) (× 50) COX-1 immunostaining of colorectal cancer and normal colonic mucosa, respectively. COX-1 expression is restricted to some stromal cells and its expression is observed in neither the cancer nor the normal epithelium. (**F**) (× 50), (**G**, **H** and **I**) (× 100) Tumours classified as grade 4, 3, 2, and 1, respectively. (**J**) (× 100) Normal colonic mucosa and mononuclear cells that we used as internal built-in controls. Normal colonic epithelium shows a very weak but diffuse staining pattern, whereas mononuclear cells are stained strongly

3 μm-thick sections. The sections were deparaffinized, hydrated through xylene and alcohol, and microwaved in Target Retrieval Solution (DAKO Japan, Kyoto, Japan) for 15 min. After washing three times in PBS, endogenous peroxidase was blocked by immersion in 3% hydrogen peroxide for 30 min at room temperature. After washing three times in PBS, non-specific binding was blocked by incubation with 10% normal goat serum for 30 min. Then, rabbit polyclonal antibody specific for human COX-2 (IBL, Gunma, Japan) diluted 40-fold with PBS was applied and sections were incubated at 4°C overnight. After washing again three times with PBS, the specimens were incubated with biotinylated anti-rabbit immunoglobulin G (IgG) for 30 min at room temperature. After washing three times in PBS, they were incubated with avidin and biotinylated horseradish peroxidase complex, followed by three washes with PBS. Then, the immune complex was visualized by immersing the slides in 0.2 mg $ml^{-1}$ diaminobenzine and 0.03% $H_2O_2$ in PBS for 1.5 min. The sections were then counterstained with haematoxylin and mounted. Non-immunized rabbit serum was used as the negative control. As an alternative control, all the 63 specimens were simultaneously stained for COX-1 using a specific rabbit polyclonal antibody (IBL, Gunma, Japan), and exactly the same methodology applied for COX-2 immunostaining.

### Evaluation of COX-2 immunostaining

The specimens immunostained for COX-2 were evaluated independently by two blinded investigators. According to the intensity and extent of positive reaction of tumour cells, the patients were divided into grades 1–4. Grades 1 and 2 were classified as low-COX-2 group, and grades 3 and 4 as high-COX-2 group. In brief, the reactions of normal colonic epithelium and stromal mononuclear cells in the colonic mucosa distant from cancer tissue were used as the internal built-in controls. Normal colonic epithelium showed a very weak but diffuse staining pattern, whereas mononuclear cells stained strongly. Immunostaining of cancer tissues was stronger than the normal epithelium in all cases. Therefore, the intensity of staining was graded on a scale of 1–4 using mononuclear cells as the reference. Grades 1 and 2 were cases with COX-2 expression weaker than, grade 3 similar to, and grade 4 stronger than, mononuclear cells. The extent of immunostaining was diffuse in all cases, and heterogeneity was observed in only a few cases.

### Statistical analysis

The correlation between patient's age or tumour size and COX-2 expression was studied using Student's *t*-test. The other clinicopathological features were analysed in relation to COX-2 expression by chi-squared test. The Kaplan–Meier method was used to estimate the survival rates without tumour recurrence, and the log-rank test was used to compare survival rates in the two groups. To determine significant prognostic factors, multivariate analysis was performed using the Cox proportional hazards modelling of factors potentially related to disease-free survival. Differences were considered statistically significant at $P < 0.05$.

### RESULTS

### Immunostaining

In all cases, COX-2 expression was predominantly localized in tumour cells, whereas some stromal cells, endothelial cells, and colonic normal mucosa adjacent to the cancer tissue stained weakly. In cancer cells, COX-2 expression was observed mainly in the cytoplasm and nuclear envelope. In general, normal colonic mucosa distant from the cancer tissue showed little or no COX-2 expression (Figure 1).

As described in Materials and methods, the patients were divided into low- and high-COX-2 groups according to the grade and extent of COX-2 expression. High COX-2 expression was

*© 2000 Cancer Research Campaign* 

**Table 1** Correlation between COX-2 expression and clinicopathological features

| Variable<br>Number of cases | | High-COX-2<br>13 | Low-COX-2<br>50 | *P* value |
|---|---|---|---|---|
| Age (yrs) | | 61 ± 9 | 61 ± 11 | 0.94[b] |
| Sex | Male | 7 | 31 | 0.59[a] |
| | Female | 6 | 19 | |
| Tumour size (mm) | | 41 ± 13 | 41 ± 25 | 0.98[b] |
| Tumour location | Colon | 5 | 31 | 0.13[a] |
| | Rectum | 8 | 19 | |
| Histological type | Well diff. Adenoca. | 8 | 39 | 0.22[a] |
| | Moderately or poorly diff.adenoca. | 5 | 11 | |
| Depth | mp | a | 25 | 0.22[a] |
| | ss ($a_1$) | 9 | 25 | |
| ly | + | 4 | 13 | 0.73[a] |
| | – | 9 | 37 | |
| v | + | 6 | 15 | 0.27[a] |
| | – | 7 | 35 | |
| LN | + | 4 | 10 | 0.41[a] |
| | – | 9 | 40 | |
| Dukes' classification | A and B | 4 | 10 | 0.41[a] |
| | C | 9 | 40 | |
| Recurrence | + | 6 | 6 | 0.005[a] |
| | – | 7 | 44 | |
| Recurence by haematogenous metastasis | + | 5 | 4 | 0.005[a] |
| | – | 8 | 46 | |

ly: lymphatic invasion; v: venous invasion; LN: lymph-node metastasis. Tumour size: maximal diameter; [a]Chi-sq; [b]Student's *t*-test.

**Table 2** Results of multivariate analysis of possible prognostic factors related to disease-free survival by Cox proportional hazards model

| Variable | Hazard ratio (95% CI) | Unfavourable/favourable | *P* value |
|---|---|---|---|
| Age (yrs) | 0.940 (0.863–1.023) | | 0.1538 |
| Sex | 0.382 (0.084–1.746) | Male/Female | 0.2148 |
| Histological type | 1.118 (0.813–1.539) | Poorly or moderately/Well | 0.4197 |
| Tumour size (mm) | 1.020 (0.991–1.050) | | 0.1795 |
| ly | 2.215 (0.542–9.054) | Positive/Negative | 0.2681 |
| v | 2.134 (0.633–7.189) | Positive/Negative | 0.2213 |
| Dukes' stage | 4.222 (0.999–17.835) | C/A and B | 0.0501 |
| COX-2 expression | 10.086 (1.971–51.612) | High/Low | 0.0055 |

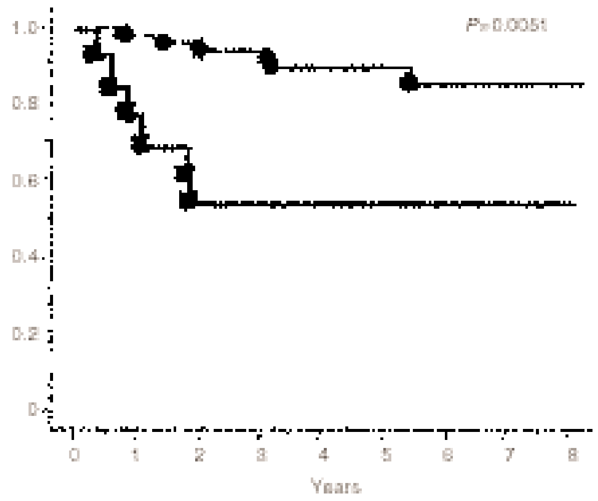

**Figure 2** Kaplan–Meier disease-free survival curves of patients in high-COX-2 (■) and low-COX-2 (●) groups. A statistically significant difference was observed between the two groups (*P* = 0.0051)

observed in 13 (21%) cases (grade 3 in 11 (18%) and grade 4 in 2 (3%)). Low COX-2 expression was observed in 50 (79%) cases (grade 1 in 20 (32%) and grade 2 in 30 (47%)).

In all cases COX-1 expression was restricted to some stromal cells and its expression was observed in neither the cancer nor the normal epithelium (Figure 1).

### Relation between COX-2 expression and clinicopathological features

In Table 1, the association between COX-2 expression and the clinicopathological features is shown. There was no significant correlation between COX-2 expression and age, sex, tumour size, tumour location, histological type, depth of invasion, lymphatic or venous invasion, lymph-node metastasis and Dukes' classification. However, high COX-2 expression was strongly correlated with tumour recurrence, especially haematogenous metastasis (*P* = 0.005).

 © 2000 Cancer Research Campaign

### Correlation of COX-2 expression with disease-free survival

The survival rate without tumour recurrence for high- and low-COX-2 groups was assessed by the Kaplan–Meier method and compared by log-rank test (Figure 2). The disease-free survival rate was significantly different between the two groups ($P = 0.0051$). Table 2 shows the results of multivariate analysis for the 63 patients. Among the 8 potentially prognostic factors (age, sex, histological type, tumour size, lymphatic invasion, venous invasion, Dukes' stage, and COX-2 expression), COX-2 expression was recognized as the only independent significant factor related to disease-free survival ($P = 0.0055$).

### DISCUSSION

So far COX-2 has been thought to play a causal role in colorectal tumorigenesis. A null mutation of COX-2 caused a marked reduction in the number and size of intestinal polyps in a murine model of FAP (familial adenomatous polyposis) $APC^{\Delta 716}$ knockout mice (Oshima et al, 1996). In a separate study, SC-58635, a selective inhibitor of COX-2, reduced the formation of aberrant crypt foci and inhibited both the incidence and multiplicity of colon tumours in the intestines of azoxymethanetreated rats (Reddy et al, 1996; Kawamori et al, 1998). Recently, it has been reported that in nude mice, selective inhibition of COX-2 by SC-58125 reduced growth of a human colon cancer cell line, HCA-7, which expresses a high level of COX-2, but it did not reduce the growth of HCT-116 cells, which lack COX-2 protein (Sheng et al, 1997). In addition, it has been shown that COX-2 level was significantly higher in colorectal tumours with larger size and in those with deeper invasion (Fujita et al, 1998). These results suggest an important role of COX-2 in colorectal tumourigenesis and growth.

Another recent report demonstrated that human colon cancer (Caco-2) cells permanently transfected with a COX-2 expression vector acquired increased invasiveness, with increased activation of matrix metalloproteinase-2 (MMP-2) and increased RNA level of membrane-type matrix metalloproteinase-1 (MT-MMP-1) (Tsuji et al, 1997). In a previous study we have also demonstrated that a selective COX-2 inhibitor, JTE-522, prevented haematogenous metastasis of colon cancer in mice (Tomozawa et al, 1999). These results suggest that COX-2 increases the metastatic potential of colorectal cancer cells.

In the present study, using immunohistochemistry, we examined COX-2 expression in advanced human colorectal carcinomas and demonstrated that COX-2 overexpression, although not correlated with the other clinicopathological features, was significantly associated with tumour recurrence, especially haematogenous metastasis.

Contrary to our results, in a recent study (Fujita et al, 1998), elevated COX-2 levels were not correlated with metastasis. In that study, however, only patients with a very short follow-up period were included, and consequently only synchronous metastasis could be evaluated. In the present study, we used samples from patients who received curative resection and had a longer follow-up (median 60 months), and consequently more reliable data on the correlation of COX-2 expression and the development of haematogenous metastasis could be obtained. Moreover, different from the previous report, in which COX-2 expression was investigated at the mRNA level and expressed as the COX-2 & COX-1 ratio, we investigated it at the protein level. These facts may explain the discrepancy between the two studies.

Haematogenous metastasis of colorectal cancer develops through a complicated process: cancer cells detach from the primary site and reach the vasculature by proteolytic cleavage of the surrounding interstitial tissues. Then the cells reach the secondary sites by adhesion to and transmigration through the endothelial cell layer and invasion of the secondary organ, and they proliferate at secondary sites by induction of angiogenesis (Liotta et al, 1986). From our data, COX-2 overexpression in colorectal cancer did not correlate with venous permeation, but with increased haematogenous metastasis formation. COX-2 expression was recognized as the only independent significant factor related to disease-free survival. Therefore, COX-2 may affect not only the proliferative and invasive properties but also the adhesion of colorectal cancer cells to extracellular matrix (ECM) protein or endothelial cells. It has been reported that rat intestinal epithelial (RIE) cells permanently transfected with a COX-2 expression vector oriented in the sense (RIE-S) demonstrated increased adhesion to ECM and expressed undetectable E-cadherin, which is involved in cell–cell adhesion (Tsujii et al, 1995). However, more studies are necessary to elucidate the effect of COX-2 on adhesion of cancer cells to ECM or endothelium, and such studies are ongoing in our laboratory.

Another possible role of COX-2 in the development of haematogenous metastasis is promotion of angiogenesis. Tumour growth and metastasis require the development of new vessels, i.e. angiogenesis (Folkman, 1990; Mahadevan and Hart, 1990; Horak et al, 1992). In a recent report, COX-2 was reported to modulate the production of angiogenic factors by colon cancer cells, while COX-1 was associated with angiogenesis regulation in endothelial cells (Tsujii et al, 1998).

Furthermore, COX-2 expression has recently been reported to be up-regulated not only in colorectal neoplasms but also in gastric, breast, oesophageal, pancreatic and lung carcinoma (Ristimaki et al, 1997; Hida et al, 1998; Hwang et al, 1998; Zimmermann et al, 1999; Tucker et al, 1999), and COX-2 overexpression enhanced lymphatic invasion and metastasis of human gastric carcinoma (Murata et al, 1999). And another recent report has shown that elevated COX-2 expression has a poorer prognostic significance in early-stage primary resected lung adenocarcinoma (Achiwa et al, 1999). These findings suggest that COX-2 may provide a new therapeutic target for several kinds of neoplasms.

To our knowledge, this is the first report on the correlation between COX-2 overexpression and recurrence of colorectal cancer, especially haematogenous metastasis, and this fact suggest that selective COX-2 inhibitors can be useful chemopreventive agents not only for tumorigenesis but also for haematogenous metastasis of colorectal cancer.

### ACKNOWLEDGEMENTS

This study was supported partly by a Grant-in-Aid for Scientific Research from the Ministry of Education, Science, Sports and Culture of Japan and partly by a grant from the Ministry of Health and Welfare of Japan.

*© 2000 Cancer Research Campaign* 

### REFERENCES

Achiwa H, Yatabe Y, Hida T, Kuroishi T, Kozaki K, Nakamura S, Ogawa M, Sugiura T, Mitsudomi T and Takahashi T (1999) Prognostic significance of elevated cyclooxygenase 2 expression in primary, resected lung adenocarcinomas. *Clin Cancer Res* **5**: 1001–1005

DuBois RN, Awad J, Morrow J, Roberts LJ 2nd and Bishop PR (1994) Regulation of eicosanoid production and mitogenesis in rat intestinal epithelial cells by transforming growth factor-alpha and phorbol ester. *J Clin Invest* **93**: 493–498

Eberhart CE, Coffey RJ, Radhika A, Giardiello FM, Ferrenbach S and DuBois RN (1994) Up-regulation of cyclooxygenase 2 gene expression in human colorectal adenomas and adenocarcinomas. *Gastroenterology* **107**: 1183–1188

Folkman J (1990) What is the evidence that tumours are angiogenesis dependent? [editorial]. *J Natl Cancer Inst* **82**: 4–6

Fujita T, Matsui M, Takaku K, Uetake H, Ichikawa W, Taketo MM and Sugihara K (1998) Size- and invasion-dependent increase in cyclooxygenase 2 levels in human colorectal carcinomas. *Cancer Res* **58**: 4823–4826

Giovannucci E, Rimm EB, Stampfer MJ, Colditz GA, Ascherio A and Willett WC (1994) Aspirin use and the risk for colorectal cancer and adenoma in male health professionals [see comments]. *Ann Intern Med* **121**: 241–246

Giovannucci E, Egan KM, Hunter DJ, Stampfer MJ, Colditz GA, Willett WC and Speizer FE (1995) Aspirin and the risk of colorectal cancer in women [see comments]. *N Engl J Med* **333**: 609–614

Hida T, Yatabe Y, Achiwa H, Muramatsu H, Kozaki K, Nakamura S, Ogawa M, Mitsudomi T, Sugiura T and Takahashi T (1998) Increased expression of cyclooxygenase 2 occurs frequently in human lung cancers, specifically in adenocarcinomas. *Cancer Res* **58**: 3761–3764

Horak ER, Leek R, Klenk N, LeJeune S, Smith K, Stuart N, Greenall M, Stepniewska K and Harris AL (1992) Angiogenesis, assessed by platelet/endothelial cell adhesion molecule antibodies, as indicator of node metastases and survival in breast cancer. *Lancet* **340**: 1120–1124

Hwang D, Scollard D, Byrne J and Levine E (1998) Expression of cyclooxygenase-1 and cyclooxygenase-2 in human breast cancer. *J Natl Cancer Inst* **90**: 455–460

Jones DA, Carlton DP, McIntyre TM, Zimmerman GA and Prescott SM (1993) Molecular cloning of human prostaglandin endoperoxide synthase type II and demonstration of expression in response to cytokines. *J Biol Chem* **268**: 9049–9054

Kargman SL, O'Neill GP, Vickers PJ, Evans JF, Mancini JA and Jothy S (1995) Expression of prostaglandin G/H synthase-1 and -2 protein in human colon cancer. *Cancer Res* **55**: 2556–2559

Kawamori T, Rao CV, Seibert K and Reddy BS (1998) Chemopreventive activity of celecoxib, a specific cyclooxygenase-2 inhibitor, against colon carcinogenesis. *Cancer Res* **58**: 409–412

Kujubu DA, Fletcher BS, Varnum BC, Lim RW and Herschman HR (1991) TIS10, a phorbol ester tumour promoter-inducible mRNA from Swiss 3T3 cells, encodes a novel prostaglandin synthase/cyclooxygenase homologue. *J Biol Chem* **266**: 12866–12872

Liotta LA, Rao CN and Wewer UM (1986) Biochemical interactions of tumour cells with the basement membrane. *Annu Rev Biochem* **55**: 1037–1057

Mahadevan V and Hart IR (1990) Metastasis and angiogenesis. *Acta Oncol* **29**: 97–103

Marnett LJ (1992) Aspirin and the potential role of prostaglandins in colon cancer. *Cancer Res* **52**: 5575–5589

Marnett LJ (1995) Aspirin and related nonsteroidal anti-inflammatory drugs as chemopreventive agents against colon cancer. *Prev Med* **24**: 103–106

Murata H, Kawano S, Tsuji S, Tsuji M, Sawaoka H, Kimura Y, Shiozaki H and Hori M (1999) Cyclooxygenase-2 overexpression enhances lymphatic invasion and metastasis in human gastric carcinoma. *Am J Gastroenterol* **94**: 451–455

O'Banion MK, Sadowski HB, Winn V and Young DA (1991) A serum- and glucocorticoid-regulated 4-kilobase mRNA encodes a cyclooxygenase-related protein. *J Biol Chem* **266**: 23261–23267

Oshima M, Dinchuk JE, Kargman SL, Oshima H, Hancock B, Kwong E, Trzaskos JM, Evans JF and Taketo MM (1996) Suppression of intestinal polyposis in Apc delta716 knockout mice by inhibition of cyclooxygenase 2 (COX-2). *Cell* **87**: 803–809

Reddy BS, Rao CV and Seibert K (1996) Evaluation of cyclooxygenase-2 inhibitor for potential chemopreventive properties in colon carcinogenesis. *Cancer Res* **56**: 4566–4569

Ristimaki A, Honkanen N, Jankala H, Sipponen P and Harkonen M (1997) Expression of cyclooxygenase-2 in human gastric carcinoma. *Cancer Res* **57**: 1276–1280

Sano H, Kawahito Y, Wilder RL, Hashiramoto A, Mukai S, Asai K, Kimura S, Kato H, Kondo M and Hla T (1995) Expression of cyclooxygenase-1 and -2 in human colorectal cancer. *Cancer Res* **55**: 3785–3789

Sheng H, Shao J, Kirkland SC, Isakson P, Coffey RJ, Morrow J, Beauchamp, RD and DuBois RN (1997) Inhibition of human colon cancer cell growth by selective inhibition of cyclooxygenase-2. *J Clin Invest* **99**: 2254–2259

Thun MJ, Namboodiri MM and Heath CW Jr (1991) Aspirin use and reduced risk of fatal colon cancer [see comments]. *N Engl J Med* **325**: 1593–1596

Thun MJ, Namboodiri MM, Calle EE, Flanders WD and Heath CW Jr (1993) Aspirin use and risk of fatal cancer [see Comments]. *Cancer Res* **53**: 1322–1327

Tomozawa S, Nagawa H, Tsuno N, Hatano K, Osada T, Kitayama J, Sunami E, Nita ME, Ishihara S, Yano H, Tsuruo T, Shibata Y and Muto T (1999) Inhibition of haematogenous metastasis of colon cancer in mice by a selective COX-2 inhibitor, JTE-522. *Br J Cancer* **81**: 1274–1279

Tsujii M and DuBois RN (1995) Alterations in cellular adhesion and apoptosis in epithelial cells overexpressing prostaglandin endoperoxide synthase 2. *Cell* **83**: 493–501

Tsujii M, Kawano S and DuBois RN (1997) Cyclooxygenase-2 expression in human colon cancer cells increases metastatic potential. *Proc Natl Acad Sci USA* **94**: 3336–3340

Tsujii M, Kawano S, Tsuji S, Sawaoka H, Hori M and DuBois RN (1998) Cyclooxygenase regulates angiogenesis induced by colon cancer cells [published erratum appears in *Cell* 1998; **94**(2): following 271]. *Cell* **93**: 705–716

Tucker ON, Dannenberg AJ, Yang EK, Zhang F, Teng L, Daly JM, Soslow RA, Masferrer JL, Woerner BM, Koki AT and Fahey TJ 3rd (1999) Cyclooxygenase-2 expression is up-regulated in human pancreatic cancer. *Cancer Res* **59**: 987–990

Williams CS and DuBois RN (1996) Prostaglandin endoperoxide synthase: why two isoforms? *Am J Physiol* **270**: G393–G400

Xie W and Herschman HR (1995) v-src induces prostaglandin synthase 2 gene expression by activation of the c-Jun N-terminal kinase and the c-Jun transcription factor. *J Biol Chem* **270**: 27622–27628

Zimmermann KC, Sarbia M, Weber AA, Borchard F, Gabbert HE and Schror K (1999) Cyclooxygenase-2 expression in human esophageal carcinoma. *Cancer Res* **59**: 198–204

 © 2000 Cancer Research Campaign
